# Rapid Return to Sport Following Focused Electrohydraulic Shockwave Therapy for a Medial Clavicle Fracture in a 14‐Year‐Old Female Soccer Player: A Case Report

**DOI:** 10.1155/cro/8427116

**Published:** 2026-09-15

**Authors:** Allen Manison, Morris Brian Polsky

**Affiliations:** ^1^ Columbia Advanced Chiropractic, LLC, Columbia, Maryland, USA; ^2^ Centers for Advanced Orthopaedics—Associates of Central Maryland Division, Columbia, Maryland, USA; ^3^ Department of Orthopaedics, University of Maryland School of Medicine, Baltimore, Maryland, USA, umd.edu

## Abstract

Clavicle fractures are common injuries in adolescents, although medial‐third fractures are relatively rare and present unique clinical considerations. This case report describes a 14‐year‐old female competitive soccer player who sustained a displaced medial clavicle fracture and was managed nonoperatively with adjunctive focused electrohydraulic extracorporeal shockwave therapy (EH‐ESWT). Treatment was initiated 4 days postinjury and administered three times per week using conservative energy parameters. The patient demonstrated rapid clinical improvement and progressive radiographic healing, achieving radiographic fracture consolidation and clearance for unrestricted return to sport at approximately 8 weeks. This recovery timeline is shorter than the 12–20 weeks reported in heterogeneous populations of clavicle fractures, although differences in fracture location, age, and study populations limit direct comparison. Although causality cannot be established from a single case, this report highlights the potential role of focused EH‐ESWT as a biologically active adjunct in fracture healing. Further research is warranted to define its efficacy, safety, and optimal application in pediatric populations.

## 1. Introduction

Clavicle fractures are among the most common skeletal injuries in both adults and children, accounting for approximately 2%–5% of all adult fractures and 10%–15% of fractures in children [1]. Within the pediatric population, clavicle fractures account for 7%–15% of all childhood fractures, with the midshaft region involved in roughly 90%–95% of cases and medial‐third injuries comprising only 2%–5% [2, 3]. Across all age groups, midshaft fractures account for approximately 80% of clavicle injuries, whereas lateral‐third fractures represent about 15% and medial‐third fractures only 5% [1]. In a large adult series, Postacchini et al. [4] reported nearly identical distributions—81% midshaft, 17% lateral, and 2% medial—and observed that midshaft injuries decrease in frequency with advancing age. The present case involves a 14‐year‐old female soccer player with a displaced medial‐third fracture, representing one of the rarest patterns of clavicle injury at an age where fracture characteristics begin to transition from pediatric to adult patterns.

The clavicle is an S‐shaped bone that connects the axial and appendicular skeleton and plays a critical role in upper extremity motion, load transmission, and protection of underlying neurovascular structures. The posterior surface also contributes to protection of vital mediastinal structures. As early as 1979, the clavicle was recognized for its role in circulation, ventilation, tension, and muscle function in the shoulder and thoracic regions [5]. The clavicle has two growth centers: the lateral and medial epiphyses. The lateral epiphysis does not fully ossify until approximately 18 years of age, and the medial epiphysis does not fuse to the diaphysis until approximately 23–25 years of age, with nearly 80% of clavicular growth occurring medially [6]. These developmental features contribute to unique fracture behavior during adolescence.

Approximately half of all clavicle fractures are sustained during sports participation [7]. Twomey‐Kozak et al. [8] evaluated athletic‐related clavicle fractures from 2015 to 2019 and found an annual injury rate of 18.72 per 100,000 at‐risk individuals, with the highest incidence among adolescents aged 10–19 years (64.5%). Football and soccer had the highest incidence of clavicle fractures.

Clavicle fractures, particularly those treated nonoperatively, typically require an extended recovery period before a safe return to sport. Waldmann et al. [9] reported that conservative management of displaced midshaft fractures results in radiographic union within approximately 18–28 weeks, with return to contact sports commonly delayed for 3–4 months. These findings align with the systematic review by Kilkenny et al. [10], which reported a mean return‐to‐play (RTP) interval of 3.35 months (103 days), with variation based on fracture location, displacement, and sport demands. Because soccer is considered a contact sport [11], this timeframe is clinically appropriate.

This case report describes the clinical course, radiographic progression, and return to sport of a 14‐year‐old competitive female soccer player who sustained a displaced medial clavicle fracture and was treated with focused electrohydraulic extracorporeal shockwave therapy (EH‐ESWT). Radiographic fracture consolidation and orthopedic clearance for unrestricted sports participation were achieved in approximately 8 weeks, a timeline that is shorter than the mean RTP interval of 103 days reported in a recent systematic review of clavicle fractures [10]. This case highlights the potential role of EH‐ESWT as a biologically active adjunct to fracture healing in a rare and clinically challenging pediatric injury. There is limited literature describing adjunctive biologic therapies to accelerate fracture healing in pediatric clavicle injuries.

This case report was prepared in accordance with the CARE (CAse REport) guidelines.

The clinical course, treatment progression, and return‐to‐sport timeline are summarized in Table 1.

**Table 1:** Timeline of clinical events.

Day	Clinical event
0	Injury sustained during a high school soccer match. Emergency department evaluation confirmed a displaced medial clavicle fracture. Sling prescribed.
3	Orthopedic evaluation confirmed a displaced medial clavicle fracture. Nonoperative management recommended with an anticipated recovery period of at least 12 weeks.
4	Initiation of focused electrohydraulic extracorporeal shockwave therapy (EH‐ESWT).
15	Follow‐up radiographs demonstrated interval healing. Continued clinical improvement noted.
21	The patient reported ability to raise arm overhead and perform activities of daily living, including securing her hair.
31	Follow‐up radiographs demonstrated progressive fracture healing.
50	Mild interval separation observed following premature backpack use and projection‐related variability cannot be excluded.
57	Eighteenth and final EH‐ESWT treatment completed.
59	Follow‐up radiographs demonstrated fracture consolidation. Orthopedic evaluation confirmed mechanical stability, and the patient was cleared for an unrestricted return to sport.
Approximately 1 year	Clinical follow‐up indicated the patient remained asymptomatic without recurrent pain, functional limitation, or fracture‐related complications. No follow‐up radiographs were obtained.

## 2. Case Presentation

**Figure 1:**
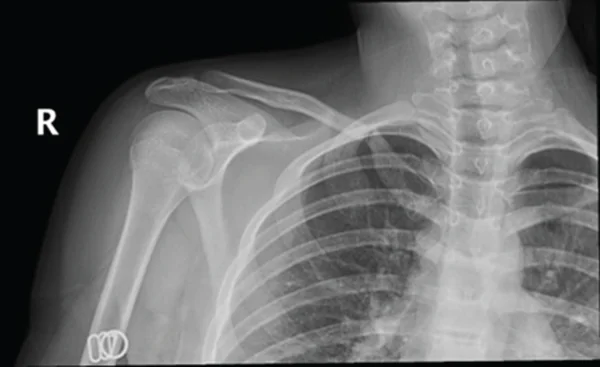
Day 0 radiographs demonstrating a proximally angulated right medial clavicular shaft fracture obtained in the emergency department.

A 14‐year‐old competitive female soccer player presented for evaluation on Day 4 following injury after sustaining a right medial clavicle fracture during a high school soccer match. The patient reported sprinting toward the ball when she fell directly onto her right shoulder. She immediately heard an audible “crack,” experienced severe shoulder girdle pain, and was unable to continue play. She was transported to a local emergency department on Day 0, where radiographs demonstrated a proximally angulated medial clavicular shaft fracture (Figure 1). The patient was placed in a sling and advised to follow up with orthopedic care.

**Figure 2:**
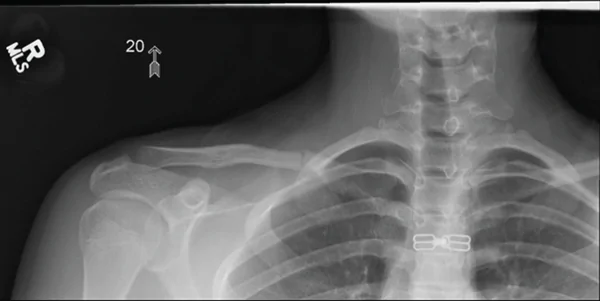
Day 3 radiographs confirming a displaced medial clavicle fracture obtained at initial orthopedic evaluation.

On Day 3, the patient was evaluated by an orthopedist (family practice physician with orthopedic fellowship training). Repeat radiographs confirmed the displaced medial clavicle fracture (Figure 2). Surgical intervention was not recommended, and a projected recovery time of at least 12 weeks was discussed with the patient and her mother.

Because the family sought a potential adjunctive option to support recovery, the patient was referred for extracorporeal shockwave therapy (ESWT). Focused electrohydraulic ESWT (StemWave Modus F device; [12]) was initiated on Day 4. Given the absence of published protocols for acute pediatric medial clavicle fractures, a conservative treatment approach was selected. Low‐energy settings (0.05–0.06 mJ/mm^2^) were chosen because of the patient′s age, the acute nature of the injury, and the proximity of the medial clavicle to underlying mediastinal and pulmonary structures. Treatment frequency and dosing were selected to provide repeated biologic stimulation while maintaining a conservative treatment approach and allowing adequate recovery between sessions.

Treatment was initially administered three times per week, delivering 1500 shockwaves per session at an energy flux density (EFD) of 0.05–0.06 mJ/mm^2^ and a frequency of 4 Hz. As the patient′s clinical and radiographic progression remained favorable, treatment frequency was reduced to two sessions per week, whereas all other treatment parameters remained unchanged.

Treatment was applied directly over the fracture site and extended medially and laterally along the clavicle to stimulate adjacent periosteal and soft‐tissue regions while avoiding excessive transmission toward the lung field.

**Figure 3:**
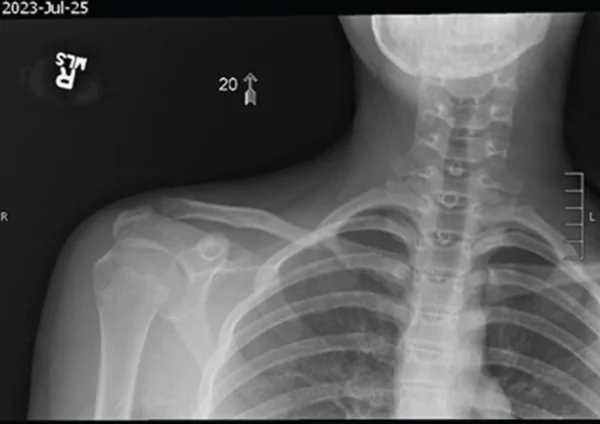
Day 15 radiographs demonstrating radiographic findings consistent with early callus formation and interval healing.

Following the first treatment, the patient reported a subjective reduction in pain. By the fifth treatment, she demonstrated improved shoulder motion and was able to abduct the arm, which she had initially been unable to do. Follow‐up radiographs obtained on Day 15 demonstrated radiographic changes consistent with early callus formation and interval healing (Figure 3).

**Figure 4:**
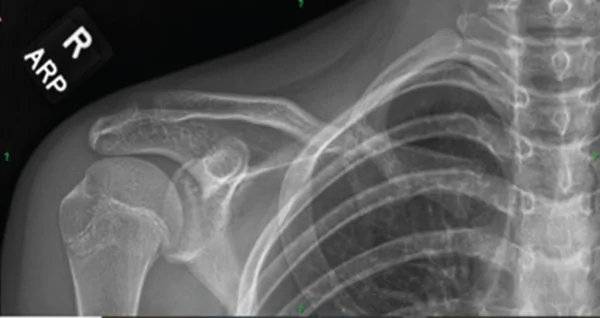
Day 31 radiographs demonstrating progressive callus maturation at the medial clavicle fracture site.

By the eighth treatment (Day 21), the patient reported being able to raise her arm overhead and perform activities of daily living, such as securing her hair. Continued clinical improvement was noted over subsequent treatments. Repeat radiographs obtained on Day 31 demonstrated progressive radiographic healing with continued callus maturation (Figure 4).

**Figure 5:**
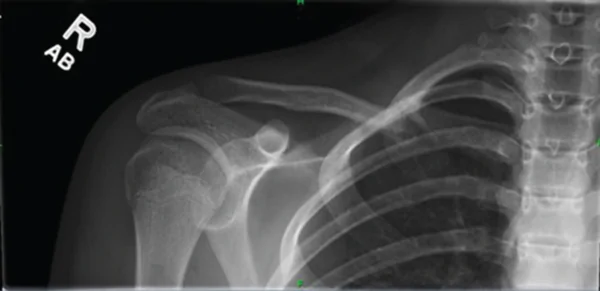
Day 50 radiographs demonstrating mild interval separation at the fracture site following reported premature backpack use. Differences in radiographic projection, positioning, and magnification cannot be excluded as contributing to the apparent interval change.

After the sixteenth treatment (Day 50), the patient reported being pain‐free, including the ability to lie on her right side without discomfort. However, interim radiographs demonstrated mild interval separation at the fracture site (Figure 5). On further questioning, the patient reported wearing a heavy backpack over both shoulders due to the absence of pain. Backpack use was considered a possible contributor to the apparent interval change; however, differences in radiographic projection, positioning, and magnification between facilities could not be excluded. The patient was instructed to discontinue backpack use immediately. This finding also highlights that symptom resolution does not necessarily indicate complete biomechanical healing, emphasizing the importance of continued activity restrictions despite substantial clinical improvement.

**Figure 6:**
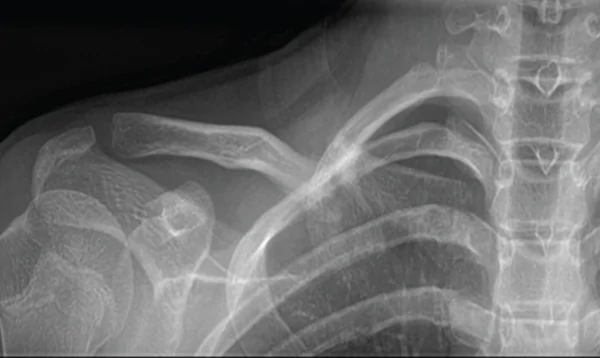
Day 59 radiographs demonstrating robust and complete callus formation with fracture consolidation prior to clearance for full return to sport.

By the eighteenth treatment (Day 57), the patient again reported improvement in comfort and function with strict compliance to activity precautions. She was referred for re‐evaluation by a second orthopedist because the original orthopedist advised that institutional policy required a minimum of 12 weeks before return to sport for displaced clavicular fractures, regardless of radiographic progression. Radiographs obtained on Day 59 demonstrated robust and complete callus formation with fracture consolidation (Figure 6). At that visit, the orthopedist integrated imaging findings with palpation of the fracture site, shoulder range‐of‐motion assessment, and functional strength testing to determine readiness for return to sport. The patient was cleared for full, unrestricted return to sport, and the orthopedist noted that the fracture appeared mechanically stable and that the patient′s risk of reinjury was no greater than that of an uninjured clavicle. No pain was elicited during examination, and the patient demonstrated full functional use of the shoulder without limitation.

A 1‐year follow‐up radiograph was not obtained. At approximately 1 year following injury, the patient remained asymptomatic without recurrent pain, functional limitation, reinjury, or need for additional treatment based on clinical follow‐up.

## 3. Discussion

This case describes an early return to sport following focused EH‐ESWT in a 14‐year‐old competitive soccer player with a displaced medial clavicle fracture. Radiographic fracture consolidation and unrestricted orthopedic clearance were achieved at approximately 8 weeks following injury. This timeline appears to be among the earliest reported returns to full sport following a pediatric medial‐third clavicle fracture treated with adjunctive focused EH‐ESWT.

ESWT is believed to exert biologically active effects through mechanotransduction, microcavitation, and upregulation of angiogenic and osteogenic mediators, including VEGF, eNOS, and BMP‐2 [13, 14]. These mechanisms have been associated with enhanced tissue perfusion and biological processes involved in bone repair. In vitro evidence suggests greater osteoblast proliferation and differentiation following EH exposure compared with other generator types [15].

Although ESWT has been investigated for fracture healing, much of the published literature has focused on delayed unions, nonunions, or surgically managed acute fractures rather than uncomplicated acute fractures. As summarized by Wang [14], the existing fracture literature varies considerably with respect to fracture type, patient population, treatment parameters, and study design, limiting direct comparison across studies. An updated Cochrane systematic review concluded that the current evidence supporting ultrasound and ESWT for acute fractures remains limited, with low‐ to very‐low‐certainty evidence for most patient‐important outcomes. The authors emphasized that additional high‐quality randomized controlled trials are needed before definitive conclusions regarding effectiveness can be drawn [16].

Furthermore, the clavicle‐specific literature, including the study by Mittermayr et al. [17], primarily involved delayed union or nonunion rather than acutely treated pediatric fractures. Consequently, direct comparison between the available fracture‐healing literature and this nonoperatively managed pediatric medial clavicle fracture is limited. Accordingly, the observations reported in this case should be interpreted with appropriate caution and should be considered hypothesis‐generating rather than confirmatory.

Because medial clavicle fractures are located near the sternoclavicular joint, mediastinal structures, brachial plexus, and lung apex, safety was an important consideration when selecting treatment parameters. Conservative energy settings were chosen, and treatment was directed along the clavicle while avoiding excessive transmission toward underlying thoracic structures. No treatment‐related adverse events, respiratory symptoms, neurovascular complications, or other complications were observed during the treatment period or follow‐up.

Medial clavicle fractures are uncommon and present distinct clinical challenges due to their proximity to the sternoclavicular joint and mediastinal structures [18, 19]. These injuries are often associated with higher energy mechanisms and may demonstrate more complex or prolonged healing trajectories. In adolescents, fracture behavior is further influenced by the presence of an unfused medial epiphysis, contributing to variable recovery patterns.

In this case, ESWT was initiated early using conservative energy parameters. The patient demonstrated steady clinical improvement and progressive radiographic callus formation. A brief apparent radiographic setback was observed on Day 50. Premature backpack use was considered a possible contributor, although projection‐related variability could not be excluded, underscoring the continued importance of mechanical protection during early healing even in the absence of pain. After strict activity modification, subsequent imaging demonstrated robust fracture consolidation.

Various clinical studies have reported favorable outcomes with focused ESWT across a variety of fracture settings. Wang et al. [20] investigated acute long‐bone fractures, Ramon et al. [21] and Moretti et al. [22] evaluated stress fractures, Quadlbauer et al. [23] studied postoperative scaphoid nonunion healing, Ahmed et al. [24] evaluated fresh mandibular fractures, and Haffner et al. [25] examined delayed tibial nonunion.

Bone fracture healing requires a highly coordinated regenerative process that involves an initial inflammatory reaction, recruitment of mesenchymal stem/stromal cells (MSCs), stimulation of osteoblastic activity, and control of osteoclasts. During the early inflammatory phase, macrophages transition from a proinflammatory (M1) phenotype to a reparative (M2) phenotype, creating a regenerative microenvironment that supports progression to the reparative phase. Experimental evidence suggests that ESWT may facilitate this macrophage polarization across multiple tissues, providing one potential mechanism by which shockwave therapy may augment normal fracture healing [26]. Osteoclasts, which arise from the monocyte/macrophage lineage, participate in coordinated bone remodeling by resorbing damaged bone, whereas osteoblasts generate new bone, thereby restoring normal skeletal architecture [27].

Experimental evidence has further suggested that appropriate ESWT energy densities enhance structural bone repair by increasing callus formation, improving bone mineral density, and promoting new bone formation during cortical bone remodeling [28]. Collectively, these findings indicate that ESWT influences multiple stages of fracture healing rather than acting through a single biologic pathway.

Previously published fracture‐healing studies have reported a wide range of EFDs, including 0.62 mJ/mm^2^ [20], 0.41 mJ/mm^2^ [23], 0.40 mJ/mm^2^ [25], 0.35 mJ/mm^2^ [24], 0.21 mJ/mm^2^ in stress fractures [21], and 0.09–0.17 mJ/mm^2^ in resistant stress fractures [22], suggesting that successful fracture healing can be achieved across a broad range of treatment parameters. Individual treatment sessions typically delivered 2000–6000 shockwaves. In contrast, the patient in the present case report was treated during the early stages of fracture healing three times weekly, with 1500 shockwaves per session, and the treatment frequency was subsequently reduced to twice weekly while maintaining an EFD of 0.05–0.06 mJ/mm^2^.

The lower energy protocol was selected because of the proximity of the medial clavicle to the lung apex, with treatment directed over the fracture and adjacent periosteal tissues while minimizing unnecessary energy transmission to the underlying lung.

Although no conclusions regarding optimal dosing can be drawn from a single case, this observation raises the possibility that successful fracture healing may depend less on maximizing delivered energy than on applying sufficient energy to stimulate the desired biologic response at the appropriate stage of fracture healing.

Because no established ESWT dosing protocol exists for acute pediatric medial clavicle fractures, the treatment frequency and total number of 18 sessions used in this case were empirically selected based on clinical judgment and the patient′s clinical progression rather than a biologically established dosing regimen.

More recently, Yoon et al. [29] evaluated early repeated ESWT following intramedullary nailing for hip fractures, administering three treatments on postoperative days 3, 7, and 11. Although ESWT was directed toward postoperative abductor soft‐tissue injury rather than the fracture site and the study was not designed to evaluate accelerated fracture union, no ESWT‐related adverse events were reported. These findings provide clinically relevant evidence regarding the feasibility and safety of initiating repeated ESWT early in the postoperative period, but do not establish an osteogenic effect in acute fractures.

Jing et al. [27] proposed a comprehensive mechanistic framework explaining how focused ESWT promotes fracture healing through sequential physical, chemical, and biologic processes. At the physical level, the shockwave generates positive and negative pressures that produce cavitation through the formation and implosion of microscopic bubbles, generating localized mechanical forces that increase cellular membrane permeability and biologic molecule ionization. At the chemical level, these mechanical effects regulate transmembrane ion channels, increase intracellular calcium flux, and convert the extracellular mechanical stimulus into intracellular biochemical signaling. At the biologic level, these intracellular signals promote angiogenesis, preserve the osteogenic potential of MSCs, stimulate osteoblast differentiation, regulate osteoclast activity, and ultimately facilitate coordinated bone remodeling.

Collectively, these sequential physical, chemical, and biologic processes provide a mechanistic explanation for how focused ESWT may enhance fracture healing across the inflammatory, reparative, and remodeling phases. In the present case, focused electrohydraulic ESWT was initiated 4 days following injury, during the early inflammatory phase of fracture healing. It is plausible that the biological effects associated with focused EH‐ESWT may have influenced the clinical course. However, the observed clinical improvement, radiographic progression, and unrestricted return to sport occurred during the period in which focused EH‐ESWT was administered; the contribution of ESWT to these outcomes cannot be determined from this single case.

## 4. Limitations

This report describes the outcome of a single patient and therefore cannot be generalized to all pediatric patients with medial clavicle fractures. Individual biological variability, natural pediatric healing potential, and fracture‐specific factors may have contributed to the favorable recovery observed in this case. In addition, this case utilized a single focused electrohydraulic ESWT device (StemWave) and a specific treatment protocol; results may differ with other shockwave technologies, energy settings, or dosing schedules. Although radiographic and clinical outcomes were favorable, controlled comparative studies are necessary to determine the true efficacy, optimal timing, and ideal dosing parameters of EH‐ESWT for acute pediatric fractures.

Formal pain scales, validated functional outcome measures, and standardized range‐of‐motion measurements were not routinely collected during clinical management and therefore were unavailable for analysis.

Follow‐up radiographs were obtained at multiple facilities as part of routine clinical care and were not acquired using a standardized research imaging protocol. Consequently, minor differences in projection, positioning, and magnification were present between studies. Radiographic findings were interpreted in conjunction with orthopedic evaluations, imaging reports, and the patient′s clinical progression.

Radiographs were interpreted as part of routine clinical care by the treating orthopedists and/or board‐certified radiologists, depending on where the imaging was performed. No independent blinded review or formal radiographic scoring system was used. In addition, no predefined radiographic criteria were utilized to classify callus formation, maturation, or fracture consolidation.

Published RTP timelines cited for comparison were derived from heterogeneous clavicle fracture populations and may not fully reflect outcomes specific to adolescent medial‐third clavicle fractures.

## 5. Conclusion

This case report describes the use of focused EH‐ESWT as an adjunct to nonoperative management of a displaced medial clavicle fracture in a 14‐year‐old competitive soccer player. The patient achieved radiographic fracture consolidation and orthopedic clearance for an unrestricted return to sport at approximately 8 weeks postinjury. Although causality cannot be established from a single case, the observed clinical and radiographic findings support further investigation of focused EH‐ESWT as an adjunct to nonoperative fracture management. These findings remain hypothesis‐generating, and further prospective studies are warranted to define its role, safety, and efficacy in pediatric populations.

## Funding

No funding was received for this manuscript.

## Ethics Statement

Ethical approval was not required for this single case report in accordance with institutional guidelines.

## Consent

Written informed consent for publication was obtained from the patient′s parent/guardian.

## Conflicts of Interest

The authors declare no conflicts of interest.

## Supporting information


**Supporting Information** Additional supporting information can be found online in the Supporting Information section. File S1: Completed CARE checklist for this case report.

## Data Availability

The data that support the findings of this study are available from the corresponding author upon reasonable request.
